# **Clinical characteristics and response to therapy of autoimmune hepatitis in an urban Latino population**

**Published:** 2016

**Authors:** Ayesha Zahiruddin, Abtin Farahmand, Paul Gaglio, Hatef Massoumi

**Affiliations:** 1 *Montefiore Medical Center, USA*; 2 *Albert Einstein College of Medicine, USA*; 3 *New York Associates in Gastroenterology and Montefiore Medical Center, USA*

**Keywords:** Autoimmune hepatitis, Hispanic patients, AIH

## Abstract

**Aim:**

We hypothesized that AIH outcomes might be different in our patient population that consists of a large number of Latinos.

**Background:**

Literature has suggested that the presentation and outcome of autoimmune hepatitis can be different among different ethnicity and communities.

**Patients and methods:**

We performed a retrospective chart review of Latino patients with AIH diagnosed between 2002-2012. Complete and partial remissions were defined as normalization of liver enzyme values, or achieving less than twice the upper limit normal (ULN), respectively.

**Results:**

A total of 28 patients were identified. 26 (93%) were female. 13 (46%) had an acute presentation, one with type 2 AIH and 3 with ANA seronegative disease. The average pathologic stage (Ishak score) was 3.44±1.67 (range: 0-6). Complete and partial remission was achieved in 20 (71%) and 5 (18%) patients respectively. Ten patients (38%) required maintenance prednisone either alone (2), or in combination with Azathioprine (6) or Mycophenolate Mofetil (2). Remission in the majority of patients, including 14 (50%) who were cirrhotic. Six of 14 (43%) cirrhotic patients were asymptomatic at the time of diagnosis.

**Conclusion:**

In an urban Latino population, cirrhosis was the initial presentation of AIH in a significant percentage of patients raising concerns regarding insufficient screening for AIH in this patient population. A large number of patients required continuous prednisone to avoid relapse.

## Introduction

 Autoimmune hepatitis (AIH) is a chronic inﬂammatory liver disease, which predominantly affects females (1,2). It is typically characterized by peri-portal inflammation consisting of plasma cells, hypergammaglobulinemia, elevated autoantibodies such as anti-nuclear antibody and anti-smooth muscle antibody, and usually responds to therapy with immunosuppression (3,4). However, none of these features are pathognomonic and there may be overlap with other liver diseases including autoimmune biliary disorders (5). Its incidence and prevalence has been reported to range between 0.15-3.0 and 11.6-35.9 per 100,000, respectively ^1^. AIH is a polygenic and multifactorial disease with numerous triggers (3). Various HLA DRB alleles have been shown to be associated with different susceptibilities to the disease in different ethnicities and races (3). AIH has variable modes of presentation and is generally an asymptomatic disease. At least a third of patients, regardless of the mode of presentation, have histological evidence of cirrhosis at the time of diagnosis (1). Patients with AIH may experience standard complications of chronic liver disease and cirrhosis. However, the rate of hepatocellular carcinoma may be diminished when compared to patients with chronic viral hepatitis (6). Prolonged therapy of autoimmune hepatitis with immunosuppressive agents remains the standard of care. However, recurrence rates in individuals who have discontinued therapy have been reported in 80% of patients (2, 7). Although the remission rate and survival in AIH is promising with immunosuppressive therapy, outcomes, natural history and response to therapy remain incompletely characterized in non-Caucasian patients. Recent studies point to advanced disease at presentation and worse outcomes in these patient populations, potentially implying a genetic component related to pathogenesis and natural history of the disease (8). Moreover, an increased disease burden of AIH in the US in a non-Caucasian patient population is likely to occur given the rapid rise in this patient population. Hispanics are the fastest growing minority in the United States. Census data predict doubling of the Hispanic patient population to encompass 30% of the US population by 2050 (9). The aim of this retrospective observational study was to assess the presentation, natural history and response to therapy of AIH in our large Latino patient population. 

## Patients and Methods

This study was approved by the Institutional Review Board approval at the Einstein Human Research Protection Program (HRPP)/Montefiore Medical Center. We performed a retrospective chart review of all Latino patients diagnosed with AIH between 2002-2012, who were treated in the Gastroenterology and Hepatology outpatient practices of the Montefiore Medical Center and New York Associates in Gastroenterology. Demographic information, as well as laboratory and liver biopsy results at the time of diagnosis were analyzed. Patient’s clinical course, including the response to immunosuppressive therapy was reviewed. The diagnosis of autoimmune hepatitis was achieved using previously published simplified criteria based on the presence and level of autoantibodies, level of serum immunoglobulin G (IgG), the absence of history of alcohol consumption and viral hepatitis, as well as compatible or typical histological features (10, 11). Liver biopsy was performed for all patients to confirm the diagnosis of autoimmune hepatitis and rule out alternative causes of elevated liver enzymes. As there is no disease specific histologic classification of necroinflammatory changes for AIH, we used the Ishak (modified Knodell) scoring system to describe the six stages of fibrosis (12). Cirrhosis was defined as stage 5 and 6. 

All patients were followed clinically, using serial measurements of liver enzymes. We defined full remission as normalization of AST and ALT. We also defined partial remission as improvement of AST and ALT to levels of twice upper limit normal. Unfortunately, most of our patients did not have a longitudinal assessment of serum gama-globulin while on treatment, and their response to treatment could only be assessed by liver enzymes. Type 1 AIH is characterized by the presence of antinuclear antibody (ANA) and anti smooth muscle antibody (SMA) and type 2 AIH is characterized by the presence of anti liver-kidney-microscome (LKM). Patients with seronegative autoimmune hepatitis do not exhibit typical autoimmune markers such as ANA, ASMA or anti LKM antibodies. However, the liver biopsy is highly suggestive of AIH. We defined acute hepatitis as elevation of AST and ALT above 10 times upper limit normal, with jaundice or acute liver failure (11). 

## Results

A total of 28 patients [mean age: 49±11 (SD) (range: 20-68); female: 26 (93%)] were identified. Acute hepatitis was the initial mode of presentation in 13 (46%) patients. Type 1 AIH characterized by the presence of ANA and anti-SMA was seen in 23 patients, type 2 characterized by the presence of anti-LKM antibody was observed in one and sero-negative disease in three patients. Liver biopsies were reviewed in all of our patients as part of the evaluation of their liver disease. We observed that the average pathologic stage (Ishak score) was 3.44±167 (SD) (range: 0-6), indicative of significant fibrosis at the time of presentation in the majority of our patients. All patients with abnormal lab tests and significant histologic injury were treated; patients were started on prednisone 40 mg per day and azathioprine 50 mg per day or prednisone 60 mg per day if they were not started on azathioprine when initiating treatment. The intended goal of therapy was to achieve normal liver enzymes. Complete and partial remissions were achieved in 20 (71%) and 5 (18%) patients, respectively. The average time from initiation of therapy to remission was 12.1±10.5 (SD) months (range 2-40). Due to the multiple problematic side effects associated with steroids, we attempted to discontinue prednisone in all patients. However, we could not discontinue prednisone in 10 patients (38%); 2 were on prednisone alone, 6 in combination with azathioprine and 2 in combination with mycophenolate mofetil (table-1). Three patients continued to have significantly elevated liver enzymes at the time of data analysis and did not achieve the primary endpoint of complete or partial remission. In addition, the population studied included 14 individuals (50%) who were cirrhotic, including 6 of 14 (43%) patients who were asymptomatic at the time of diagnosis. 

## Discussion

Over the last decade, multiple aspects of AIH have been elucidated, including appropriate diagnostic criteria, serological markers, pathogenesis, and treatment (9). However, the effect of ethnicity on the natural history of autoimmune hepatitis (AIH) has not been well studied. Our retrospective analysis identified a high frequency of Cirrhosis (50%) at presentation in this Urban Latino patient population with AIH. The frequency of cirrhosis was high in both symptomatic and asymptomatic patients. Although, 89 percent of patients achieved either complete or partial remission within one year, 38 percent of them needed to be on steroids to avoid relapse. This indicates that in this patient population, AIH posed a serious health risk based on both disease severity and potential risks associated with long term treatment with steroids. 

Literature suggests that AIH in non-white patients may present more frequently with advanced disease at initial diagnosis, rapid progression to cirrhosis and more advanced clinical features including cholestasis (9). In one study, frequency of cirrhosis at presentation was significantly greater in African Americans than in Whites (13). In two different studies published by Wong RJ, et al. (14) and Munoz-Elpinosa L (15), cirrhosis at presentation was observed in up to 56% of Hispanics. Our data add to this growing body of literature, which indicates that AIH may be more problematic in a Hispanic/non-Caucasian patient population, indicating that efforts should be made to educate health care providers to screen for this disease in minority patient populations. 

**Table 1:** Basic patient’s characteristics and treatment response

No	Age	Cirrhosis	Acute	Pathology Grade	Pathology Stage	Medication	Remission	Time to remission (month)	Note
1	67	_	+	U	U	A	+	4	
2	36	+	+	4	6	P-M	+	9	Allergic reaction to azathioprine
3	55	_	_	1	4	A	+	4	Seronegative, repeat biopsy showed stage 1 after 18 month of therapy
4	31	+	+	4	4	P-M	_	9	Severe leukopenia with azathioprine
5	52	_	+	3	2	P-A	+	4	
6	45	+	+	4	4	A	+	6	
7	64	_	+	3	2	P-A	_	U	4+ positive AMA, disease is still active by laboratory findings
8	56	_	_	3	2	B-A	+	11	On budesonide for mild flare
9	52	_	_	3	2	A	+	2	
10	42	_	_	U	1	A	_	U	4+ positive AMA, disease is still active by laboratory findings
11	50	+	_	2	6	A	+	14	
12	40	+	_	2	5	A	+	4	
13	52	+	+	2	2	M-B	+	18	
14	42	_	_	2	2	B	_	6	Allergic reaction to azathioprine
15	36	+	+	U	U	A	_	7	Multiple flares
16	57	+		1	6		+	U	Biopsy showed cirrhosis, no detailed information available
17	36	+	_	U	6	P	U	U	Overlap AIH and PSC
18	46	+	+	1	3	M-B	+	16	Aseptic osteonecrosis of bone due to steroids
19	68	+	+	4	3	P-A	+	5	Steroid induced diabetes
20	20	+	_	3	3	A	+	36	
21	51	_	_	U	U	M	+	3	Azathioprine induced severe bone marrow toxicity
22	55	+	+	3	0	P-A	+	4	Lupus glomerulonephritis
23	48	_	_	2	4	P-A	+	12	
24	49	_	_	3	3	P-A	+	26	Hepatitis C, cured with PEG-interferon and ribavirin AST stayed elevated after HCV therapy, responded to azathioprine
25	44	_	+	4	4		+	10	
26	51	_	+	3	2	A	+	28	
27	55	+	_	3	4	M	+	40	Allergic reaction to azathioprine
28	65	+	_	U	6	P	U	U	

A: azathioprine, B:budesonide, M:mycopehonolate mofetil, P:prednisone, U:unknown

We have identified that response to therapy was attenuated in our patient population. Lim KN, et al. noted that AIH tends to present at a younger age and is more advanced in African Americans compared with white patients. Despite this late presentation, the disease responds well to immunosuppressive therapy. However, greater doses of prednisone were required to maintain remission, and the prognosis for African Americans with AIH may be worse than it is for white patients.^13^ Prospective studies are needed to better define the natural history of AIH in minority patient populations. In our study, a large number of patients required maintenance prednisone to avoid relapse, potentially indicating that AIH was more difficult to treat in Latino patients. 

A significant percentage of our patients presented with cirrhosis, raising concerns regarding insufficient screening for AIH in this patient population. It has been shown that Hispanics generally have lower compliance with screening guidelines specifically related to cancer than non-Hispanic whites and non-Hispanic blacks (16). Hispanics are also less likely to have health insurance than any other racial or ethnic groups (17). In a report of insurance coverage of people under 65 living in the US in 2005, 13% of non-Hispanic Whites, 21% of non-Hispanic African Americans and 34% of Hispanics did not have health insurance and Health insurance coverage disparities persisted among employed people as well, of note, these data reflect an era prior to the Affordable Care Act (18). 

Ethnic variance in AIH epidemiology may reflect underlying genetic differences, contributing to variations in presentation, disease severity, response to therapy, and overall clinical outcomes in certain population. Examples of genetic differences related to natural history and response to therapy of other liver diseases have been previously presented, including NAFLD and PNPLA gene diversity, as well as HCV related to IL28. Our observations related to racial differences in natural history, presentation, and response to therapy of AIH in Latinos indicate that genome wide association studies (GWAS) to identify potential genetic polymorphisms that contribute to genetic differences in AIH need to be performed. In addition, further epidemiologic, patient cohort, and clinical trials should be performed in this patient population.

In conclusion, in this urban Latino patient population of patients with AIH, cirrhosis was present in a large number of patients at the time of initial presentation, including both symptomatic and asymptomatic patients. Complete or partial biochemical remission can be achieved in the majority of these patients. However, a significant number of patients could not be weaned off immunosuppression and required maintenance prednisone to avoid relapse. A significant percentage of patients presented with cirrhosis, raising concerns regarding insufficient screening for AIH in this patient population. This study has some limitations, including but not limited to the retrospective nature of the study, relatively small number of patients and lack of a comparative arm such as non-Latino whites or African Americans. However, the advanced presentation of AIH in this Latino population and inability to discontinue steroids in a significant percentage of these patients indicate that further multi-center studies are warranted in this patient population.
